# Confocal Blood Flow Videomicroscopy of Thrombus Formation over Human Arteries and Local Targeting of P2X7

**DOI:** 10.3390/ijms22084066

**Published:** 2021-04-14

**Authors:** Patrizia Marchese, Maria Lombardi, Maria Elena Mantione, Domenico Baccellieri, David Ferrara, Roberto Chiesa, Ottavio Alfieri, Chiara Foglieni

**Affiliations:** 1 Department of Immunology and Microbiology, Scripps Research, La Jolla, CA 92037, USA; patrimarchese1@gmail.com; 2 Myocardial Diseases and Atherosclerosis Unit, Cardiovascular Research Center, San Raffaele Scientific Institute IRCCS, Via Olgettina, 58, 20132 Milano, Italy; lombardi.maria@hsr.it (M.L.); mantione.mariaelena@hsr.it (M.E.M.); 3 Cardiothoracic and Vascular Department, San Raffaele Scientific Institute IRCCS, Via Olgettina, 58, 20132 Milano, Italy; baccellieri.domenico@hsr.it (D.B.); david.ferrara@sangiovannieruggi.it (D.F.); chiesa.roberto@hsr.it (R.C.); alfieri.ottavio@hsr.it (O.A.)

**Keywords:** thrombogenicity, internal mammary artery (IMA), carotid plaque (CPL), platelets, fibrin, tissue factor (TF), factor XI, P2X purinoceptor 7 (P2X7)

## Abstract

Atherothrombosis exposes vascular components to blood. Currently, new antithrombotic therapies are emerging. Herein we investigated thrombogenesis of human arteries with/without atherosclerosis, and the interaction of coagulation and vascular components, we and explored the anti-thrombogenic efficacy of blockade of the P2X purinoceptor 7 (P2X7). A confocal blood flow videomicroscopy system was performed on cryosections of internal mammary artery (IMA) or carotid plaque (CPL) determining/localizing platelets and fibrin. Blood from healthy donors elicited thrombi over arterial layers. Confocal microscopy associated thrombus with tissue presence of collagen type I, laminin, fibrin(ogen) and tissue factor (TF). The addition of antibodies blocking TF (aTF) or factor XI (aFXI) to blood significantly reduced fibrin deposition, variable platelet aggregation and aTF + aFXI almost abolished thrombus formation, showing synergy between coagulation pathways. A scarce effect of aTF over sub-endothelial regions, more abundant in tissue TF and bundles of laminin and collagen type I than deep intima, may suggest tissue thrombogenicity as molecular structure-related. Consistently with TF-related vascular function and expression of P2X7, the sections from CPL but not IMA tissue cultures pre-treated with the P2X7 antagonist A740003 demonstrated poor thrombogenesis in flow experiments. These data hint to local targeting studies on P2X7 modulation for atherothrombosis prevention/therapy.

## 1. Introduction

Atherothrombosis is a major cause of life-threatening complications, associated with vascular luminal barrier impairment by rupture/erosion/calcified nodule [1,2]. Cycles of rupture/healing without thrombosis have been observed in relation to luminal surface compromise [3,4,5], which depends on multiple, differently combined factors [6,7,8,9,10]. Localization, extension and deepness of the rupture determine the nature of sub-endothelial vascular components (either preserved in their integrity or broken) encountering unphysiological contact with blood, thus affecting thrombus formation [11].

Although arteries constitutively express pro-thrombotic molecules [12,13,14,15,16,17,18,19], and pro-coagulant/angiogenic plasma players [20], microparticles and microvesicles [21,22] may accumulate in the atherosclerotic wall, the role of vascular tissue components in the thrombus formation and potential resistance to therapy is still not completely understood. 

Coagulation mechanisms and shear-dependent platelet functions have been investigated using either parallel plate flow chambers [23,24] or microfluidic platforms [25,26,27] mimicking patho-physiological hemodynamic conditions over cultured cells or protein-coated surfaces. Flow studies have been performed over human plaque homogenates, suggesting that vascular collagen rapidly induced platelet adhesion and aggregation through activation of glycoprotein VI, while tissue factor (TF) was crucial for fibrin formation [13,28,29]. The features of sites where platelets and fibrin interact with human vascular wall and the thrombus forms remain scarcely investigated.

In this study, a confocal videomicroscopy system allowing the perfusion under controlled flow conditions of human blood over sections from human arteries was optimized to quantify and localize platelet aggregation and fibrin deposition. We compared the thrombogenicity of human atherosclerotic carotid artery plaque (CPL) and atherosclerosis-resistant internal mammary artery (IMA) [30,31], providing preliminary evidence of structure-related vascular responsivity to blood in the presence/absence of coagulation pathways inhibition. 

We previously reported that arteries express P2X purinoceptor 7 (P2X7) in association with atherosclerotic damage [32]. P2X7 was described to play a pro-thrombotic role in a murine model of ferric chloride-induced carotid artery thrombosis [33], and intragastrical pre-treatment of mice with P2X7 antagonism prevented platelets activation and thrombosis [34]. Strategies for thrombosis prevention and bleeding risk reduction are evolving [35]. Here we explored the possibility of inhibiting thrombus formation onto arterial sections by pre-treating cultured human vascular tissue ex vivo, i.e., before exposure to flowing blood, with the specific P2X7 antagonist A740003 [36]. 

## 2. Results

The workflow followed in the study is summarized in a flowchart (Supplemental Figure S1).

### 2.1. Thrombogenic Response of IMA and CPL Sections

**Figure 1:**
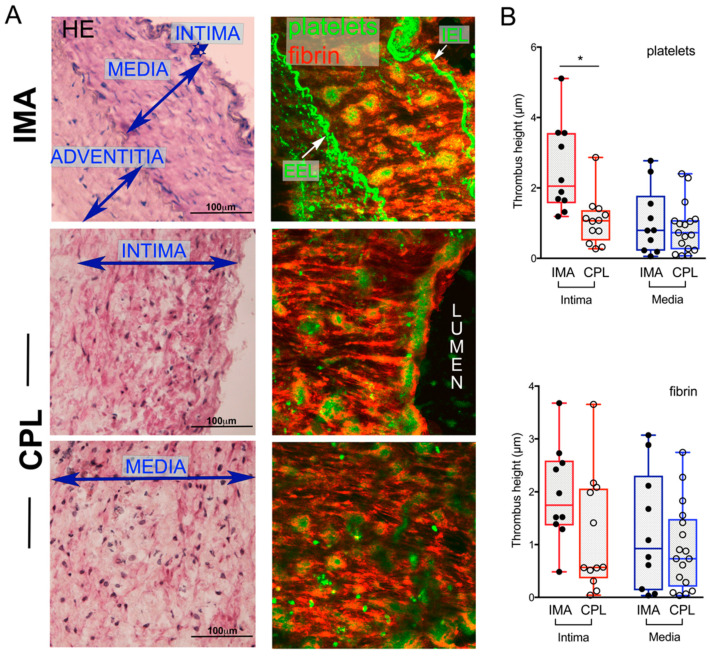
Thrombus formation over arterial tissue sections. Representative images from internal mammary artery (IMA) and carotid artery plaque (CPL) fields are shown (**A**). Histology (hematoxylin/eosin, **A** left column) and corresponding post-blood flow 2D projections from colorized Z-sections (**A** right column) show the presence of not homogeneously distributed platelet aggregates over the vascular walls. IEL: internal elastic lamina, EEL: external elastic lamina. Quantification of platelets aggregates (**B**, top) and fibrin deposition (**B**, bottom) over the layers of 10 IMA and 17 CPL in blood flow experiments are compared. Not all the layers were available for sampling in all the CPL. Data are presented as boxes (5–95 percentile) and dots indicate the mean value from 3 to 14 fields/sample; values showing thrombus height (volume/ROI area) are presented. Asterisks indicate significant differences obtained by non-parametric Kruskal–Wallis analysis. * *p* value < 0.05 is considered significant.

Flowing normal human blood over IMA and CPL sections led to the formation of thrombi over all the vascular layers (Figure 1A, Supplemental Figure S2). Thrombi displayed platelet aggregates within shells of fibrin, interconnected by fibrin strands. The internal elastic lamina fibers were not thrombogenic in both IMA and CPL, while thrombi were assessed over the neighboring tissue (not shown). 

The size of platelet aggregates was significantly different over the intima but not over the media on IMA vs. CPL; the amount of fibrin was comparable in both arteries (Figure 1B). The individual variability (Supplemental Figure S3A,B) and the donor-related difference in blood reactivity (Supplemental Figure S3C) influenced the variance.

Vascular markers, including molecules that may support thrombus formation (e.g., tissue TF, fibrin(ogen), von Willebrand Factor, collagen type I and laminin), were differently distributed in IMA and CPL layers (Table 1). P2X7 was heterogeneously observed in the regions supporting thrombus formation in CPL but not in IMA (Supplemental Figure S4).

These data suggested a possible different local interaction between blood pro-coagulant elements and vascular tissue.

### 2.2. Effects of Selective Blockade of Coagulation Pathways on Thrombogenesis over IMA and CPL 

To investigate the involvement of blood coagulation pathways in thrombus formation over vascular tissues, we analyzed whether and how cocktails of monoclonal antibodies blocking the function of circulating TF (aTF) or FXI (aFXI) influenced thrombogenesis over IMA and CPL. 

**Figure 2:**
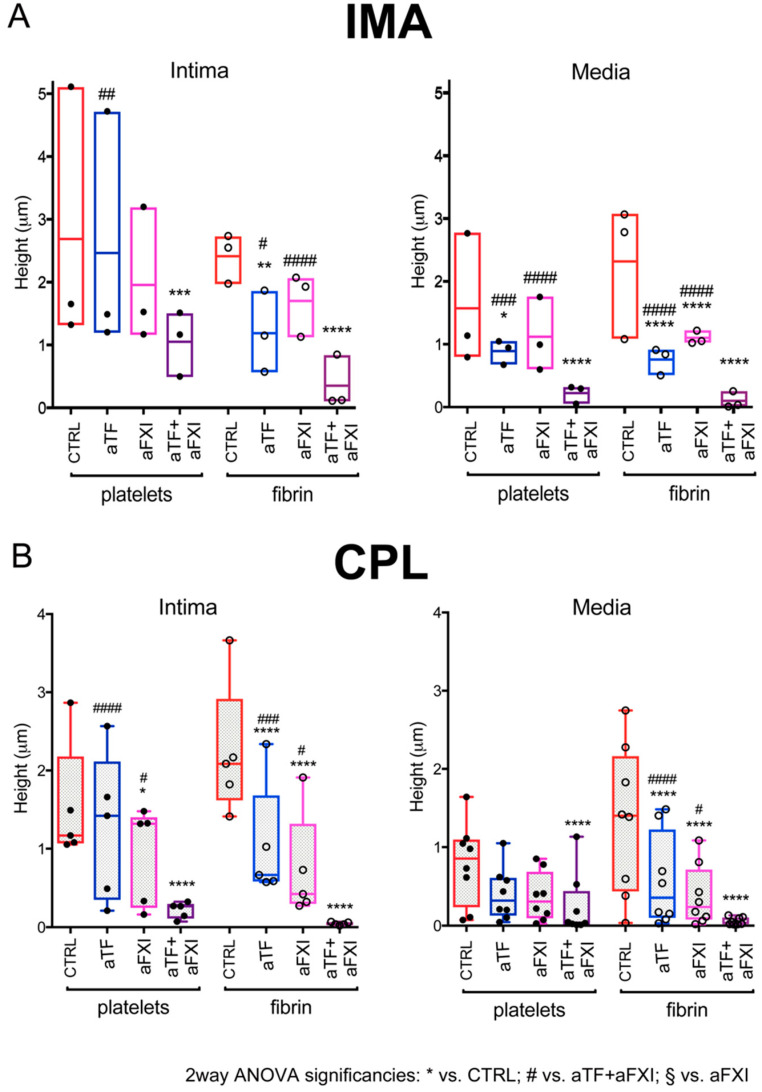
Influence of coagulation pathway blockades on thrombus formation over vascular tissue. Quantification by confocal sectioning of platelet aggregated and fibrin deposited over 3 IMA (**A**) and 8 CPL (**B**) sections upon perfusion of normal human blood. Not all the layers were available for sampling in all the CPL. Circulating TF and/or coagulation FXI were neutralized by adding specific monoclonal antibodies to blood (aTF, aFXI). Data are presented as boxes (5–95 percentile), and dots indicate the mean value from ≥4 to 5 fields/sample; thrombus height (volume/ROI) is presented. Symbols indicate significant differences: * vs. CTRL, # vs. aTF + aFXI by 2way ANOVA with Tukey’s multiple comparisons test for paired comparisons. *, # *p* < 0.05 and **, ## *p* < 0.01, ***, ### *p* < 0.001, ****, #### *p* < 0.0001.

The addition of either aTF or aFXI to blood caused partial and variable inhibition with respect to CTRL. Specifically, platelets aggregation was reduced by aTF in the media of IMA (Figure 2A right), and by aFXI in the intima of CPL (Figure 2B left). 

The combination of aTF + aFXI demonstrated higher inhibitory effect on platelets aggregation vs. aTF and vs. aFXI over intima of CPL (Figure 2B left) vs. aTF over intima and media of IMA, and vs. aFXI over media of IMA (Figure 2A). Fibrin deposition was significantly, albeit partially, inhibited by addition either of aTF or aFXI to blood and quite abolished by aTF + aFXI over vascular layers of both IMA and CPL (Figure 2).

These results showed that thrombogenesis over IMA and CPL tissues was dependent from both TF- and FXI-mediated thrombin generation for full support of platelet adhesion/aggregation and fibrin deposition.

### 2.3. Sub Analysis of CPL Media with Morphological Alterations: Effects of Selective Blockade of Coagulation Pathways on Thrombogenicity 

In the above paragraph, the values/layer for each vessel were grouped and averaged, and thus the analysis did not account for potentially dissimilar local responses due to vascular wall heterogeneity in the presence of atherosclerosis. 

To evaluate this role, we arbitrarily defined in CPL four media subtypes (see Methods) with distinct morphological abnormalities in cellular composition and extracellular matrix architecture: minimally altered media (AM), lipo-cellular media (LM), fibrotic media (FM) and necrotic core (NC). Media subtypes differed in proteoglycans (abundant in AM and LM, rare in FM and NC, Supplemental Figure S5A); expression of collagen type I, laminin, TF and fibrin(ogen) (Table 2, Supplemental Figure S5B). 

**Figure 3:**
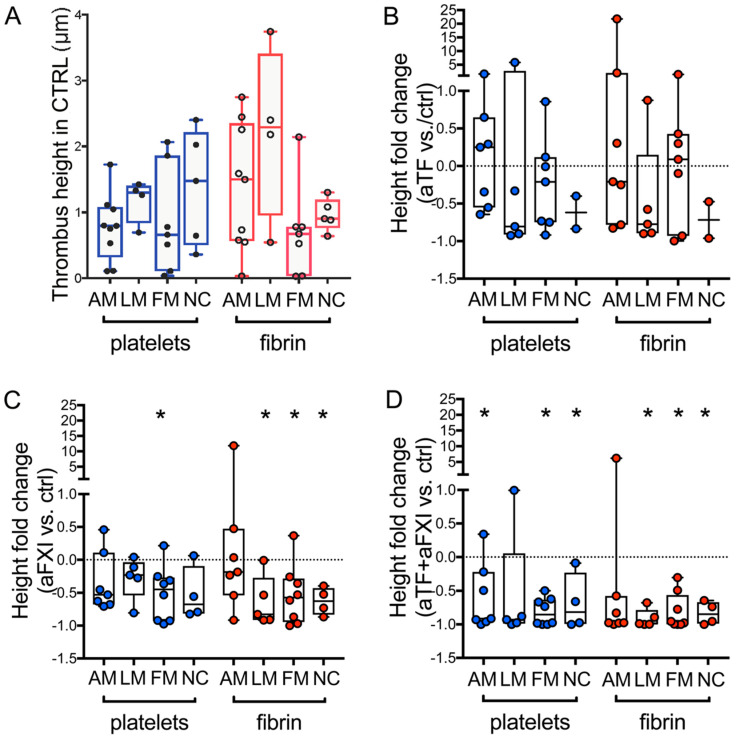
Influence of coagulation pathway blockades on thrombus formation over CPL media subtypes. Comparison of platelet aggregates (blue) and fibrin deposition (red) over the media sub-layers of 11 CPL in blood flow experiments using control blood (**A**). Thrombus formation in the presence either of aTF (**B**), or aFXI (**C**), or aTF + aFXI (**D**) is quantified in fibro-calcific areas (FM), necrotic core (NC), lipoproteins and/or foam cell-rich areas (LM), or minimally altered (AM) fields of media from 7 CPL. Degree of inhibition (fold changes in thrombus height vs. CTRL) is presented. The dashed line at 0 indicates the absence of changes. In each panel the data are displayed as boxes (5–95 percentile), and dots indicate the mean value from ≥3 fields/sample. Asterisks in (**B**–**D**) indicate significant differences in fold change vs. CTRL for each media subtype, obtained by non-parametric Wilcoxon matched-pairs signed rank test (hypothetical value for inference = 0). * *p* values < 0.05 are considered significant. Not all the subtypes are detected in all the CPL.

The average volume of platelet aggregates and fibrin clots did not vary significantly among the media subtypes in CTRL (Figure 3A). In agreement with the concept that thrombosis does not arise from fibro-calcified plaques [1] and the notion that NC could include coagulation enhancers [13], no thrombus formation was observed over areas of the FM fields with micro-calcification, scarcely cellularized and mostly marker-negative, but over NC fields displaying the presence of tissue TF and traces of collagen type I (Supplemental Figure S5A,B). 

The addition of aTF to blood did not lead to significant changes in thrombus formation over media subtypes vs. CTRL (Figure 3B), in agreement with the detection of tissue TF and P2X7 (Supplemental Figure S6).

The addition of aFXI to blood was variably effective over AM but significantly decreased platelets aggregation over FM (poor in collagen type I) and fibrin deposition over LM, FM and NC (Figure 3C). 

Nearly complete inhibition of thrombus formation was diffusely found in blood with aTF + aFXI (Figure 3D). 

A marked decrease of co-localized platelets and fibrin was found by analyzing the corresponding fields (see Methods) of media subtypes with all the antibody cocktails vs. CTRL (Supplemental Figure S7). 

### 2.4. Sub Analysis of CPL Intima with Distinct Histological Features: Effects of Selective Blockade of Coagulation Pathways on Thrombogenicity 

**Figure 4:**
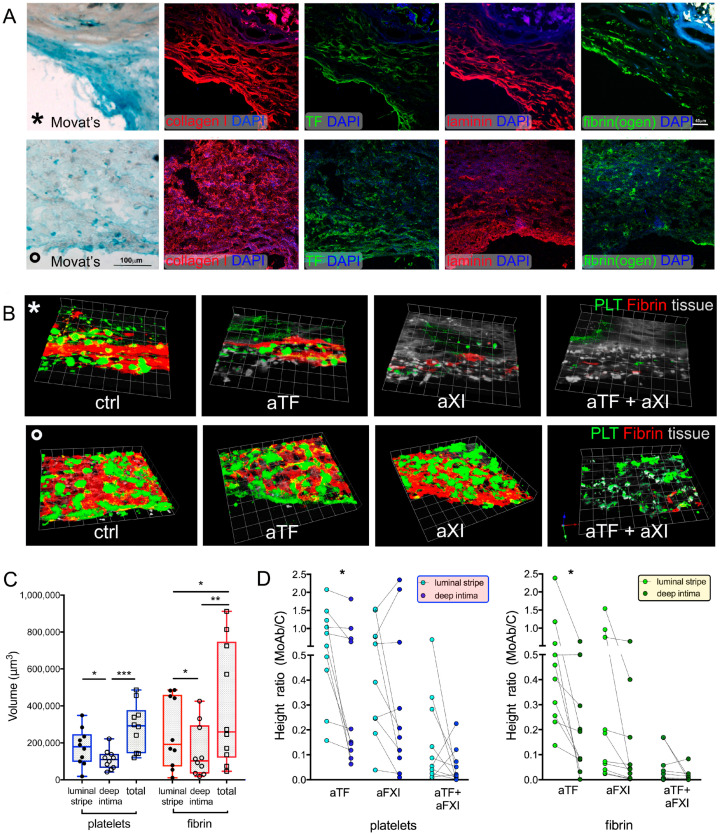
Influence of coagulation pathway blockades on thrombus formation over portions of CPL intima. Histology and distribution of immunolabelled molecules important for thrombus formation are presented (same fields of serial sections; symbols *, ° indicate the correspondence) (**A**). Three-dimensional renderings from confocal Z sections with colorized platelets and fibrin (**B**) show the global spatial changes in thrombus formation over intima in the presence of aTF and/or aFXI into blood in two selected fields from two CPL. Quantitative analysis of thrombus formation over intima of 5 CPL perfused with control blood (**C**) and with blood added with aTF and/or aFXI (**D**) shows differences between the sub-endothelial (luminal stripe, 70 μm thick) and the deeper portions (deep intima) in platelet aggregation and fibrin strand deposition. Data from 10 fields are presented as boxes (5–95 percentile) with dots indicating the thrombus volume (**C**) or as before–after dot plot with dotted lines connecting luminal stripe and deep intima of the same field (**D**). Paired *t*-test was applied. Significant differences: * *p* < 0.05, ** *p* < 0.01, *** *p* < 0.001.

The intra-sample variation in height of platelet aggregates and fibrin deposition was lower in the intima of IMA (34.3% ± 19.6 and 33% ± 9.7, respectively) than of CPL (54.4% ± 29.2 and 49.5% ± 28.2, respectively), consistent with morphological heterogeneity of CPL (Figure 4A). 

To evaluate the influence of morphology on thrombogenicity, the intima fields were divided into 2 subfields: the luminal stripe (sub-endothelial region, 70–75 µm thick [37]), and the deep intima (underlying area). 

The platelet aggregates and fibrin clots formed by CTRL blood were significantly greater in the whole field (total) than in the subfields, but only fibrin differed between luminal stripe and deep intima (Figure 4B,C, Supplemental Figure S8). 

The addition of aTF to blood was significantly less effective in the luminal stripe than in deep intima (Figure 4B,C), that of aFXI was comparable, and aTF + aFXI almost abolished thrombus formation in all subfields (Figure 4B,C, Supplemental Figure S8). 

Differences in thrombus formation appeared morphology-related; abundant proteoglycans, thick extracellular matrix fibers of high-density collagen I and laminin, tissue TF fibrin(ogen) and P2X7 were detected in the luminal stripes over where dense fibrin strands assembled and the efficacy of aTF was scarce (Figure 4A,B * rows, Supplemental Figures S5 and S6). In contrast, patchy distributed thrombi were observed in areas displaying no evident structural difference between luminal stripe and deep intima, and showing collagen type I organized in fibrils/thin fibers (curl aspect or random distribution) (Figure 4A,B ° rows).

### 2.5. Effect of Vascular Tissue Pre-Treatment with P2X7 Antagonist on Thrombogenesis

**Figure 5:**
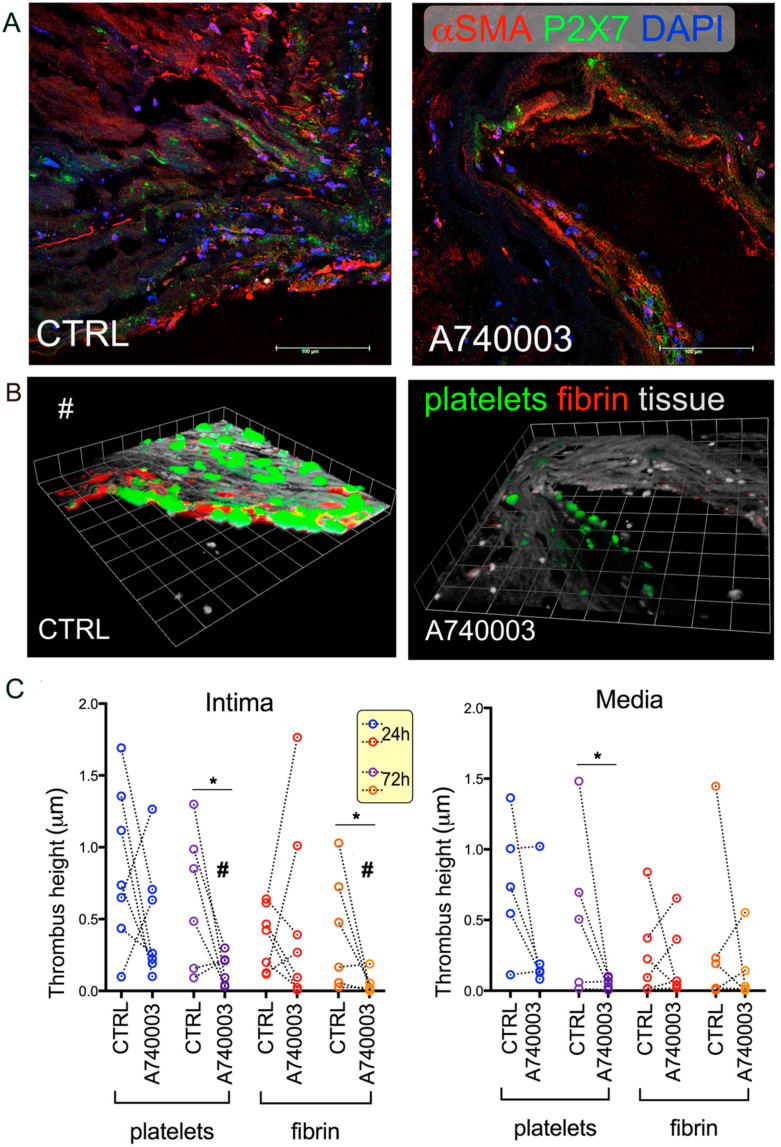
Effect of P2X7 antagonism by A740003 on thrombus formation over CPL intima and media. Representative intima fields from carotid artery plaque (CPL) in confocal images showing the expression of αSMA (red) and P2X7 (green). Nuclei are stained with DAPI (blue) (**A**). The same fields are displayed in 3D rendering images from confocal Z sections, with colorized platelets and fibrin (**B**). Quantitative analysis of thrombus formation over intima (**C**, left) and media (**C**, right) of 6 CPL pre-treated ex vivo with A740003 (t24 h and 72 h of treatment in tissue culture, respectively), sectioned and perfused with control blood shows is presented. Data from at least 5 single fields are presented as before–after dot plot with dotted lines connecting the fields with the same X, Y. The symbol # indicates the same intima field in (**A**–**C**); * *p* < 0.05 by *t*-test.

To test whether a local decrease of the tissue thrombogenicity (TF-related in the intima) of vessels was feasible, we pre-treated arteries in tissue culture with a P2X7 specific antagonist, A740003 [36], before sectioning and flow experiments (see Methods). Thrombus formation was nearly abolished over P2X7^+^ intima fields of the CPL pre-treated with A740003 for 72 h vs. not pre-treated (Figure 5A–C left). Shortest treatment (24 h) [38] led to variable responses (Supplemental Figure S8A left).

Similar behavior was shown by platelet aggregation over media of pre-treated CPL, while fibrin deposition demonstrated some variability (Figure 5C right,), compatible with P2X7 heterogeneous distribution (Supplemental Figure S8A right). 

In agreement with the poor expression of P2X7, the IMA pre-treated with A740003 failed to show relevant decreases in thrombus formation (Supplemental Figure S9A,B).

## 3. Discussion

Thrombus formation involves multiple interactions and we have studied in a fixed hemodynamic condition two players, the vascular tissue and the coagulation pathways. We show that (i) atherosclerotic CPL are not globally more thrombogenic than healthy IMA; (ii) both coagulation pathways are involved in thrombus formation over the vascular layers, and some local differences may be related to vascular structure and components; and (iii) tissue targeting of P2X7 may contribute to thrombogenesis modulation (summarized in the Graphical Abstract). 

A two-step mechanism based on platelet interaction with vascular collagen, a potent inductor of platelets aggregation [13], and on successive thrombin generation with fibrin formation driven by tissue TF but not involving blood TF [16,29] was proposed by studies on human plaque homogenates. No association between coagulation data and vascular components was achievable using homogenized samples, and our system (Supplemental Figure S2) was aimed at fulfil this gap, allowing one to quantify of thrombus formation, identify the thrombogenic regions over artery sections, and delineate their features.

Our findings, e.g., greater platelet aggregation in the intima of IMA than of CPL and comparable thrombogenesis in the media of both arteries, confirm the relationship between local presence of collagen Type I in the vascular wall and thrombogenesis. Although not unresponsive to stimuli [30,31], the grafted IMA rarely undergo thrombotic occlusion [39,40], and our findings suggest that this in vivo scarce susceptibility is not due to a poor hemostatic capability of the arterial wall but to high efficiency of the luminal barrier. 

Our data also demonstrate that blockade of a single coagulation pathway is variably efficient, and selective inhibition of both coagulation cascades into blood is required to abolish thrombus formation over IMA and CPL. 

Moreover, our data suggest a synergy between coagulation pathways in interacting with the vascular wall, possibly related to amount and heterogeneous distribution of pro-coagulant markers. 

The first layer exposed to blood by vascular damage is the intima, thin and homogeneous in IMA, heterogeneous in thickness and altered by infiltrating or migrating cells and abnormal extracellular matrix deposition in CPL [41]. Our findings demonstrate that in situ alterations of the intima locally affect thrombogenesis. By blocking the blood extrinsic pathway by aTF, thrombus formation persisted over the luminal stripe, displaying thick proteoglycans, collagen type I and/or laminin fiber bundles, which suggests that both composition and three-dimensional organization of extracellular matrix network could be implicated in thrombus formation. In vivo studies are required to validate the existence of intima portions either highly thrombogenic, or partially retaining TF-related thrombogenicity. 

In term of differences, the blockade of the blood intrinsic pathway by aFXI is more effective than aTF on fibrin in highly altered vs. less damaged media. Although in light of recent reports we cannot exclude the existence of FXI-independent thrombin generation [42], the activation of FXIa is known to generate thrombin through activation of FIX, or enhancement of prothrombotic activity of extrinsic pathway via inactivation of TFPI [43].

Translation of the blockade of coagulation cascades in a clinical benefit for patients at risk of thrombosis is puzzling, but dedicated studies taking in account the need for a balance between thrombus inhibition and increased bleeding risk are required [44,45,46,47].

Among pro-coagulant markers is tissue TF, secreted by microvesicles, smooth muscle cells, and mononuclear cells in murine and human atherosclerotic plaques [18,33]. The highest efficiency of aTF + aFXI vs. aTF in inhibiting thrombus formation over CPL and IMA may indicate that only part of the tissue TF is in the active form, and the pool of tissue (fibrin)ogen *di per se* is insufficient to trigger relevant and stable platelets aggregates in these blocking conditions. Accordingly, we preliminarily detected weak and focal signals for cryptic TF and fibrin in CPL only (data not shown). However, the modulation of thrombogenicity by inhibiting tissue TF was proposed using human vessel homogenates [48], whilst the activation of the P2X7 pathway induced TF generation by vascular cells and affected thrombosis in mice [33]. A putative therapeutic potential of P2X7 antagonism for cardiovascular diseases is emerging [34,44,49]. We previously reported the heterogeneous expression of P2X7 by human arteries and the suitability of the P2X7 antagonist A740003 as a tool to decrease inflammatory status in ex vivo CPL [32,38,50]. In the present study we tested the effectiveness of local targeting of the vascular tissues using A740003. In analogy with the recent report on entecavir in a murine model of chemically induced carotid thrombosis [34], we showed that A740003 inhibits platelets aggregation over CPL intima and media, but it is also effective on fibrin. An inhibitory effect of A74003 on P2X7^+^ platelets and platelet microvesicles already present in the vascular wall is feasible. The absence of inhibition over IMA not only is consistent with the paucity of expressed P2X7 [32] but also suggests that the antagonist has no undesirable effects on hemostasis. These results support further extensive studies on local targeting of atherothrombosis-prone vessels. Additional investigations are also required to establish a functional relationship between thrombus formation and P2X7 in human vasculature

## 4. Materials and Methods

### 4.1. Patient Samples

Carotid artery plaque fragments (CPL, n = 17) were harvested from 56 to 90 years old patients undergoing thrombo-endarterectomy because of a >70% stenosis by NASCET criteria, without ictus or transient ischemic attack of the anterior circle (OXFORD criteria). These specimens were obtained at surgery by eversion, included tunica intima and media but only the inner part of adventitia. Non-atherosclerotic internal mammary artery specimens (IMA, n = 10) were obtained from age-matched patients (n = 8) undergoing coronary artery by-pass surgery and used as controls. No patient had overt symptoms of ongoing inflammatory diseases; and all vascular specimens represented surgical waste. Baseline data of the enrolled patients are listed in Supplemental Tables S1 and S2. The study protocol conformed to the ethical guidelines of the 1975 Declaration of Helsinki and was approved by the Ethics Committee at San Raffaele Hospital; all patients signed informed consent.

### 4.2. CPL and IMA Processing 

Biopsies were collected in 0.9% NaCl saline solution with penicillin (100 UI/mL) immediately after surgery, macro-sectioned, embedded in Killik (BioOptica, Milan, Italy), and snap-frozen in 2-methyl butane/liquid N_2_. Since all specimens were surgical waste, to avoid damage or alterations biasing the results, only IMA with surrounding perivascular adipose tissue were included in the study, whilst the fragments from artery skeletonized during grafting procedure were discarded. 

Before being snap-frozen, a subset of sample rings from IMA (n = 3) and CPL (n = 6) was kept in culture, as previously described [38], and 2 rings/fragment were either untreated or submitted to treatment with A740003, added to culture medium at 100 μM for 24 h (t1) or 72 h (t3).

Numbered serial cryosections (10 μm thick) were obtained and dedicated either to confocal videomicroscopy experiments (pair numbers) or to histology and immunofluorescence (odd numbers). For blood flow videomicroscopy experiments, the sections were collected on glass coverslips coated with bovine fibronectin (50 μg/mL in phosphate buffered solution, R&D Systems Inc., Minneapolis, MN, USA). 

Reagents, whereas not differently indicated, were purchased from EuroClone S.p.A., Milan Italy and Sigma-Aldrich Saint Louis, MO, USA.

### 4.3. Histology and Immunofluorescence

Vascular sections were stained with hematoxylin/eosin and Movat’s pentachrome to assess morphological features. The presence of fibrous cap, foam cells, fibro-lipids, ulcerations, necro-thrombotic core, calcifications, hemorrhage/thrombosis and inflammatory infiltrates was determined in CPL [1,51]. Sections were observed under an Eclipse55i microscope equipped with DS-L1 camera (all from Nikon, Tokyo, Japan) and composed to obtain whole section images. Samples showing evidence of blood stasis either due to intra-plaque hemorrhage or to surgical procedure were excluded from the study to avoid a potential bias in flow experiments.

Immunofluorescent labelling followed by confocal microscopy was performed as previously described [30,32] to analyze vascular markers (Supplemental Table S3) Sections were examined under a Leica TCS-SP5-AOBS confocal microscope (Leica Microsystems GmbH, Wetzlar, Germany); free projection max images were obtained from single channel acquired Z-series and merged by Adobe PhotoshopCS. 

### 4.4. Confocal Blood Flow Videomicroscopy Experiments

Blood samples were harvested at Scripps Research’s Normal Blood Donor Service, providing normal human blood for investigators as a core service for the institute and based directly on the service provided by the Scripps General Clinical Research Center (for healthy blood donors requirements see: https://nbds.scripps.edu). According to the privacy law, protecting information about donors, data about the individuals donating blood are not available.

Blood flow experiments were performed with human blood collected in citrate phosphate dextrose solution (CPD, 11 mM citrate final concentration) and re-calcified with 10 mM CaCl_2_ immediately before perfusion over the artery section. A custom-built rectangular parallel plate flow chamber, realized in order to have a constant/homogenous shear rate in the whole chamber and hosting 25 × 50 mm coverslips was used in real-time confocal videomicroscopy (LSM450 confocal microscope, Carl Zeiss Inc., Oberkochen, Germany) to visualize deposition of platelet aggregates and fibrin strands over blood-perfused cryosections (7 min, wall shear rate of 500 s^–1^ [52]). Platelets were labelled by 10 mM mepacrine and fibrin by mouse anti-human fibrin AlexaFluor546-conjugated (clone mh1, 0.02 mg/mL, from ATCC hybridoma, which binds specifically to the β chain of fibrin and has no cross-reactivity with the β chain of fibrinogen) added to blood. 

In a set of experiments, cocktails of monoclonal antibodies against different epitopes of human Factor-XI (clones 1A6 and 14E11 at 30 μg/mL each) and/or human-TF (clones 5G9, 9C3 and 6B4, 50 μg/mL each) were added to blood 5 min prior of perfusion. All the antibodies were kind gift of W. Ruf. Setup flow studies on surfaces coated either with collagen or purified TF, performed in Ruggeri’s laboratory showed that simultaneous blockade of the epitopes, but no longer incubation time was required to maximize the inhibitory effects. 

Not influent anti-thyroglobulin antibody served as control (control blood, CTRL). No coagulation-inhibiting antibodies were added to donor’s blood in the set of experiments on sections from plaques untreated/treated with A740003.

At the end of blood perfusion, sections were rinsed with DMEM (Gibco, Life Technologies Inc, Carlsbad, CA, USA) by perfusion through the chamber at 1/5th of the initial flow rate. Stacks of confocal Z-sections were collected sequentially at 488 nm (platelets) and 543 nm (fibrin) wavelength at preset X, Y positions, with a Z-step of 2 μm, sampling different vascular layers on the base of histology analysis. 

After application of median filter by MetaMorph software (Molecular Devices, LCC, San Josè, CA, USA), a first region of interest (1st ROI) was drawn based on tissue shape, excluding areas empty or with an air bubble between slide and tissue. The 1st ROI was applied to stacks after thresholding (Huang threshold) by ImageJ (NIH) software. Threshold values were chosen to separate platelets and fibrin signals from tissue autofluorescence. Platelet aggregates and fibrin deposition were measured in the ROI. 

A sub-analysis of media fields was performed on the basis of the different morphological features. The media fields were allocated to 4 arbitrarily defined sub-layers: (1) Minimally altered media (AM) demonstrating co-presence of normal and altered features in the field of view; (2) lipo-cellular media (LM) with foam cells and/or lipoproteins in ≥50% of a field of view; (3) fibrotic media (FM) with extended fibrosis and/or calcified nodules and scarce/no cellularity; and (4) necrotic core (NC) of the atheroma with cell debris or no cells.

The intima was further analyzed by dividing each field in two areas luminal stripe and deep intima. Considering that CPL portions prone to rupture may show a fibrous cap ranging within 70–100 µm [37], to delineate the luminal stripe, a 2nd ROI large 75 μm from the luminal side but keeping the contour of that drawn for the total field was obtained. Deep intima area was obtained by subtracting luminal stripe area (2nd ROI) from the total area (1st ROI). 

Absence of potential artefacts such as thrombus formation over the glass or originating from the luminal endothelium was verified and excluded during the methodological setup. The areas where tissue detachment from the slide locally altered the laminar flow, and the regions where auto-fluorescence due to tissue elements biased the thrombus determination were excluded from the analysis, except for the intima sub-analysis where also single fields were considered, from 3 to14 fields/layer/sample were quantified. In the sub-analyses of CPL, media and intima corresponding fields were compared, i.e., fields having similar X, Y in serial sections with respect to a morphological *repere* found in all sections from the same vessel. 

The size of platelet aggregates and fibrin deposition was calculated as total volume over measured ROIs, yielding average height.

The absence of thrombus formation over the coated glass without tissue was always verified. To avoid bias due to different blood donors in experiments where antibodies against human-TF and/or factor XI were added to blood, for each field the value/field obtained in the presence of these antibodies was normalized vs. the mean value/CPL of the corresponding control blood (CTRL) supplemented with not influent antibody, then the mean value/CPL was calculated. Based on X,Y coordinates of fields in sections submitted to blood flow experiments, the corresponding regions were re-localized on sections submitted to histology and to immunofluorescence. 

Z-project images were obtained by ImageJ using the original Z-sections with tissue (grey) for sections without thrombi, and colored in green/red/yellow (=platelets/fibrin/colocalization, respectively) for sections with thrombi. In all cases of Z-sections with presence either of fibrin or platelets on tissue, a merge of grey with red or green images was used. Three-dimensional (3D) renderings were built from Z-projects using Volocity 5.0 software (Perkin-Elmer, Milano, Italy).

The method is schematized in Supplemental Figure S2.

### 4.5. Statistical Analysis

Differences in height (volume/area) values were analyzed with one-way ANOVA and Kruskal–Wallis test for multiple comparisons. Inhibition values were analyzed by two-way ANOVA with Tukey’s multiple comparisons test, or by *t*-test or non-parametric *t*-test (depending from normality of the distribution). *p* < 0.05 was considered significant. 

## 5. Conclusions

This proof-of-concept study demonstrates the suitability of a novel system for the study of in vitro thrombogenic response to normal blood of healthy IMA and an atherosclerotic CPL wall, which was efficiently inhibited by specific blockade of both coagulation pathways. The extrinsic (FXI) pathway results are mostly relevant for the media response and the intrinsic (TF) pathway for that of intima and hints at the existence of differentially thrombogenic portions. Specific antagonism on CPL tissues of the TF-related pro-thrombogenic P2X7 provides a preliminary indication in favor of thrombus formation prevention through local targeting of atherosclerotic vessel components, hinting to extensive tests using other mediators and conditions.

## 6. Study Limitations

The present study has inherent limitations. 

The blood donors used in flow experiments partially account for the variability in the entity of platelets aggregates and fibrin deposition over vascular sections. The impossibility to obtain blood samples harvested from different donors at the same time hampered the realization of normalized experiments with fresh blood pools. A wall shear rate of 500 s^–1^ [52] was tested as proof of concept; the evaluation of the shear variable over tissue layers could be the subject of a future comparative study applying different shear rates with the same/different blood.

The blood flow direction was parallel to the tissue surface, but longitudinal sectioning did not allow it to obtain homogeneously exposed luminal surface. Consequently, the intima response was studied in transversally-cut sections, discarding regions where endothelium received the flowing blood from the back. 

A limited third dimension of arterial tissue (height of the section) was allowed by the design of the parallel plate flow chamber to ensure a laminar and homogenous flow. In the presented conformation, our system was not suited for studying the influence of depth and shape of plaque disruption on thrombus formation. Moreover, the unavailability of additional tissue hampered extensive analyses of the same human plaque at different levels along the arterial length.

In our series, all patients providing vascular fragments were receiving a therapy modulating the blood coagulation. In particular, through reducing TF vascular expression [53], statins could affect the intra-layer variability in fibrin deposition. 

The limited number of specimens also impaired the establishment of a relationship between arterial reactivity (especially of IMA) and patient status. 

Finally, advanced atherosclerotic lesions represented CPL samples, and evaluation of the adventitia was discarded because only its inner part was available and potentially exposed to handling damage during surgery.

## Figures and Tables

**Table 1:** Semi-quantitative evaluation by confocal microscopy of markers expression: IMA vs. CPL.

Marker	IMA		CPL	
	Intima	Media	Intima	Media
TF (tissue factor)	+cells of the luminal lining; −/traces intima	+/traces	++cells of the luminal lining and several intima cells *	++ ‡
Fibrin(ogen)	+cells of the luminal lining; −intima	−	++ not continuous along the luminal lining; +/−intima †	+/++cells *, †
vWF (von Willebrand factor)	++ not continuous along the luminal lining; intima	−	++ not continuous along the luminal lining; −intima	traces/–
αSMA (alpha smooth muscle actin)	+intima	+++	+/++ †	+/++ †, §
SM22 (smooth muscle protein 22)	+several intima cells	+	++scattered cells	+ †
SMMHC (smooth muscle myosin heavy chain)	+intima	++	+rare cells	++scattered cells §
CD68	−	++rare cells	−; +several cells †	++rare cells §
FSP1 (fibroblast-specific protein-1)	+ not continuous along the luminal lining and/or several intima cells	+rare cells	+several intima cells	++rare cells §
Vimentin	+++ not continuous along the luminal lining and in the intima	+	+	++ §
Laminin	+++luminal lining;+intima	++	++luminal lining; +/++intima †	+/−
Collagen type I	+	+/traces	+/++	+/++ †
P2X7	traces	−/traces	+ †	+/traces/− †

**Symbol legend:** * often granular signal; † heterogeneous; ‡ cells sm22+; § fibrotic tissue.

**Table 2:** Semi-quantitative evaluation by confocal microscopy of marker expression: CPL Media subtypes.

	CPL Media Subtypes			
	AM	LM	FM	Necrotic Core
TF (tissue factor)	++ ‡; +/traces other cells *	++/− *, †	−; ++ ‡	++/− *, †
Fibrin(ogen)	++/− †	++	traces/++ *, †	traces/–
vWF (von Willebrand factor)	−	−	−	−
αSMA (alpha smooth muscle actin)	+ †	+ rare cells	−	−
SM22 (smooth muscle protein 22)	++ scattered /rare cells	+ rare cells	++rare cells	−
SMMHC (smoot muscle myosin heavy chain)	++ scattered /rare cells	+ rare cells	+rare cells	− (+ rare cells) †
CD68	+ rare cells	++ scattered cells	++rare cells	−
FSP1 (fibroblast-specific protein-1)	traces	+/traces	–	traces/−
Vimentin	+/++ †	traces/–	–	−
Laminin	+/− †	−	−	−
Collagen type I	+/++ †	+/traces †	traces/–	traces/−
P2X7	traces/−	+/traces	−	−

**Symbol legend:** * often granular signal; † heterogeneous; ‡ cells sm22+. **Acronyms:** minimally altered media (AM), lipoproteins and/or foam cells -rich media (LM), fibro-calcific media (FM), necrotic core (NC).

## Data Availability

No dataset have been deposited in repository, and the data from the studied patients are not publicly available, in agreement with privacy law and our institutional policies.

## Supplementary Materials

The following are available online at https://www.mdpi.com/article/10.3390/ijms22084066/s1.
