# Supplementary material for: Confocal Blood Flow Videomicroscopy of Thrombus Formation over Human Arteries and Local Targeting of P2X7
Source: Int J Mol Sci. 2021 Apr 14;22(8):4066. doi: 10.3390/ijms22084066 (PMC8071050; doi:10.3390/ijms22084066)
Supplement: Supplementary file 1 [file ijms-22-04066-s001.zip › Supplemental data IJMS2021.pdf]

Supplementary Materials to:

Confocal blood flow videomicroscopy of thrombus formation over human arteries and local targeting of P2X7

Patrizia Marchese PhD[1, §], Maria Lombardi MSc[2, §], Maria Elena Mantione MSc[2], Domenico Baccellieri MD[3], David Ferrara MD[3], Roberto Chiesa MD[3], Ottavio Alfieri MD[3], and Chiara Foglieni PhD[2]

[1] Department of Immunology and Microbiology, Scripps Research, La Jolla, CA, USA
[2] Myocardial diseases and Atherosclerosis Unit, Cardiovascular Research Center, San Raffaele Scientific Institute IRCCS, Milano, Italy
[3] Cardiothoracic and Vascular Department, San Raffaele Scientific Institute IRCCS, Milano, Italy

[§] equally contributing authors

Supplemental Table1. Baseline clinical characteristics of patients undergoing TEA

	**(N = 17)**
Age (years)	72±8 *
Sex (M/F)	15/2
% Stenosis	75 (70-100) **
Carotid (dx/sx)	9/8
**Major risk factors, n (%)**	
Pregressed IMA/ICTUS (>6months)	4
Hypertension	13
CAD/HF	5
PAD	3
Dyslipidemia	6
Current smokers	5 (1 ex-)
**Medications, n (%)**	
Statins	5
Antiaggregants	14
b-blockers	4
ACE inhibitors	5
Ca antagonists	0
Other	7

* Mean+SD; ** Media (range)

Supplemental Table 2. Baseline clinical characteristics of patients undergoing CABG

	**(N = 8)**
Age (years)	63±13 *
Sex (M/F)	8/0
**Major risk factors, n (%)**	
Positive family history of IMA/ICTUS	0
Hypertension	6
Dyslipidemia	4
Current smokers	2
**Medications, n (%)**	
Statins	7
Antiaggregants	7
b-blockers	6
ACE inhibitors	5
Other	4

* Range min to max

Supplemental Table 3. Antibodies/reagents used in confocal microscopy

1ary antibodies	Clone	Company	Dilution
Mouse anti-Human PECAM1/CD31	M0823	Dako, Glostrup, Denmark	1:100
Rabbit anti-Human von Willebrand factor	/	Dako, Glostrup, Denmark	1:200
Mouse anti-Human smooth muscle α-actin	1A4	R&D System Inc, Minneapolis MN	1:500
Goat-anti- SM22	/	Abnova, Taipei, Taiwan	1:200
Mouse anti-Human smooth muscle myosin heavy chain	SMMS-1	Dako, Glostrup, Denmark	1:100
Rabbit anti- FSP1/S100A4	/	Millipore, Temecula, CA	1:500
Mouse anti- CD68	KP1	Dako, Glostrup, Denmark	1:100
Mouse anti-Human collagen type I	I-8H5	Calbiochem, Darmstadt, Germany	1:100
Rabbit anti- laminin	/	Sigma-Aldrich Saint Louis, MO	1:400
Rabbit anti- Tissue Factor $_{1\text{-}218}$	/	Not commercial, kind gift of W. Ruf	1:100
Rabbit anti-Human fibrin(ogen) FITC-conjugated	/	Dako, Glostrup, Denmark	1:100
Rabbit anti-Human P2X7	/	Chemicon Europe Ltd, Chandlers Ford, UK	1:200

2ary antibodies	Company	Dilution
AlexaFluor 594 Goat anti-Mouse-IgG (H+L)	Molecular Probes - Invitrogen, Eugene, OR	1:500
AlexaFluor 488 Goat anti-Rabbit-IgG (H+L)		
AlexaFluor 680 Donkey anti-Goat-IgG (H+L)		

Nuclear stain	Company	Concentration
4',6-diamidino-2-phenylindole	Sigma Immunochemicals, St Louis, MO	0.2nM

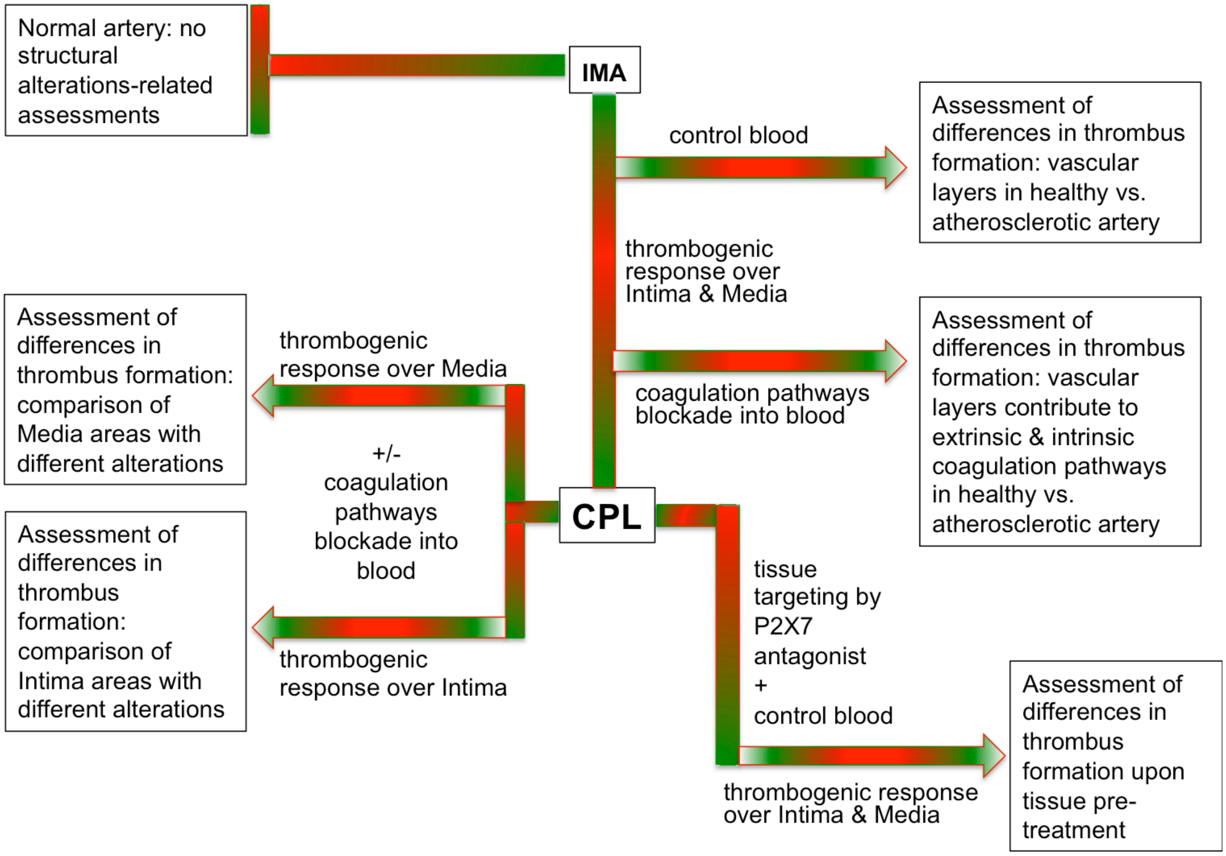

Supplemental Fig. 1 Study workflow

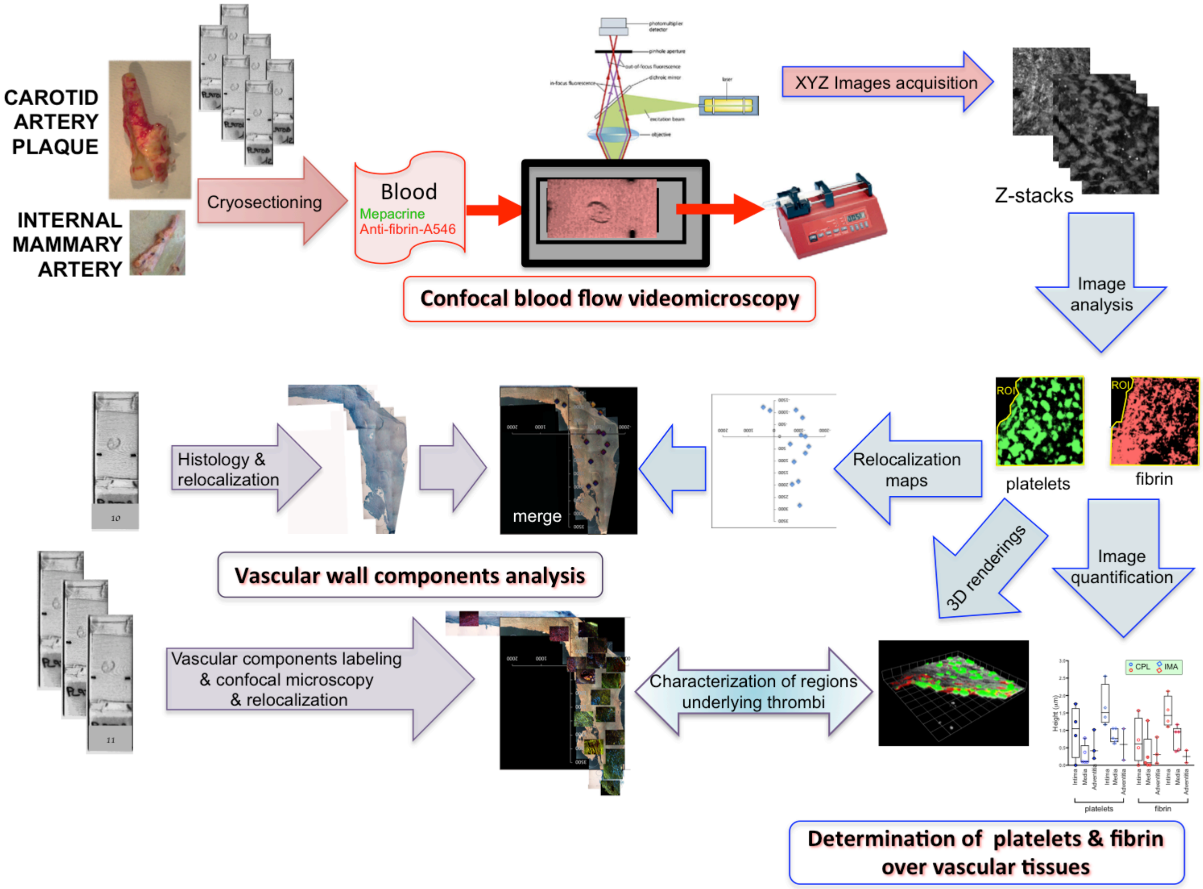

Supplemental Fig. 2 Method for the study of thrombus formation ex-vivo over arteries

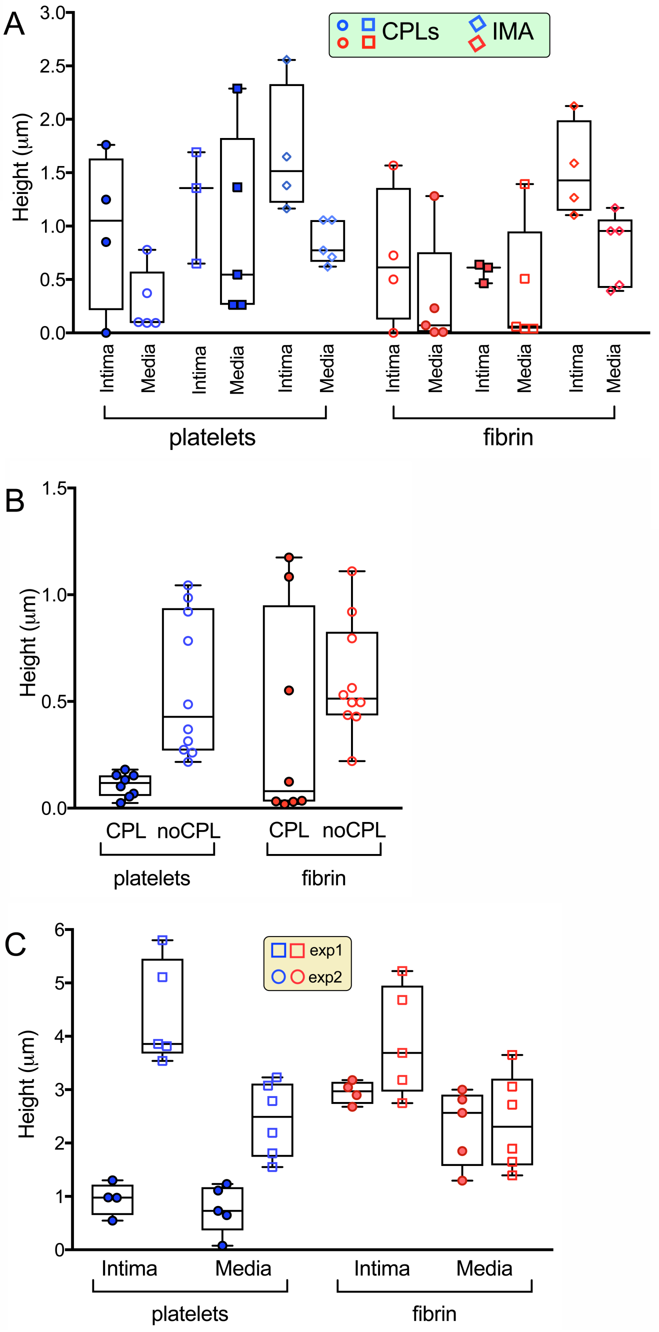

Supplemental Fig. 3 Thrombus formation over atherosclerotic and not atherosclerotic vascular beds: quality controls.

Comparisons of 2 CPL and 1 IMA perfused with blood from the same healthy donor in the same experiment (**A**); of the stenotic portion (CPL) vs. that not stenotic and far from the lesion (noCPL) belonging to the same carotid artery and perfused with blood from the same donor in the same experiment (**B**); of two blood donors on serial sections of the same CPL using control (+ anti-t**γ**, see methods) blood (**C**) are shown. Data from single fields are presented as boxes (5-95 percentile) with dots indicating the thrombus height. Non-parametric Kruskal-Wallis test was applied. Significancy: ** $p<0.01$.

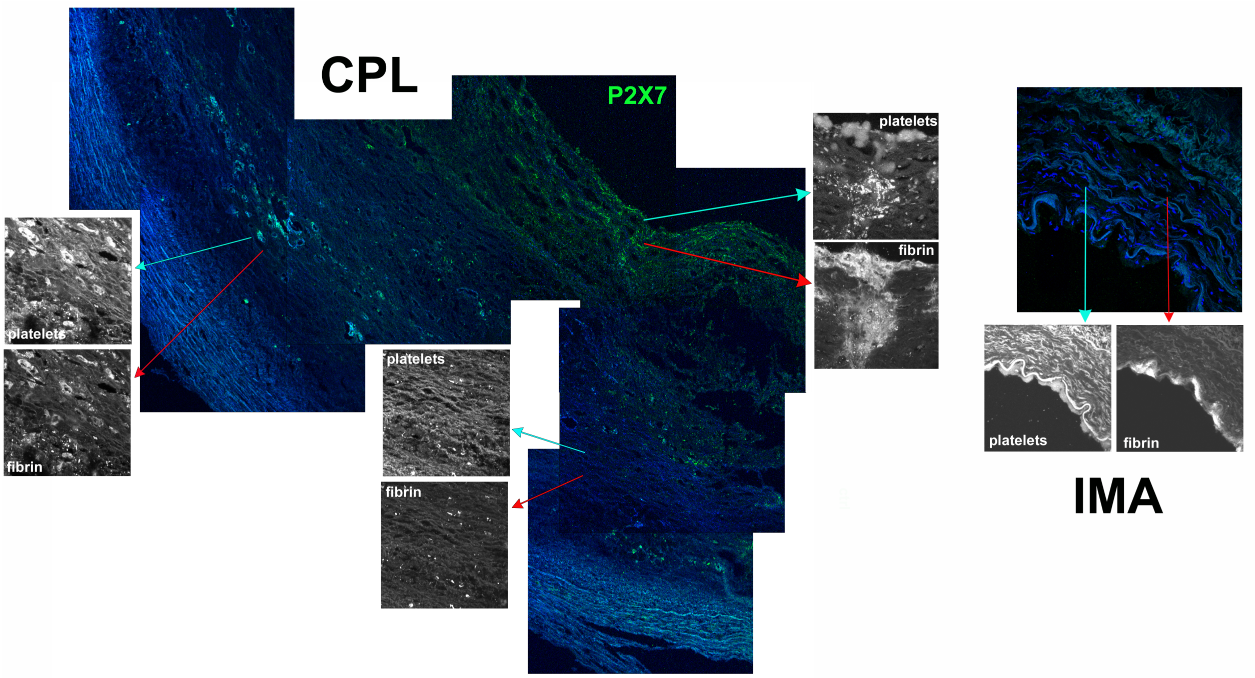

Supplemental Fig. 4 P2X7 localization and thrombus formation over CPL and IMA sections
Partial reconstruction of 1 CPL, i.e. collage of adjacent fields, and representative confocal image of 1 IMA show the distribution of P2X7 in the tissue. The images in grey display the thrombus formed (arrows) over serial sections; platelet aggregates and fibrin present in the same field are shown as separate panels. All the images are 2D projection max images from confocal microscopy Z-series acquired with objective 40x

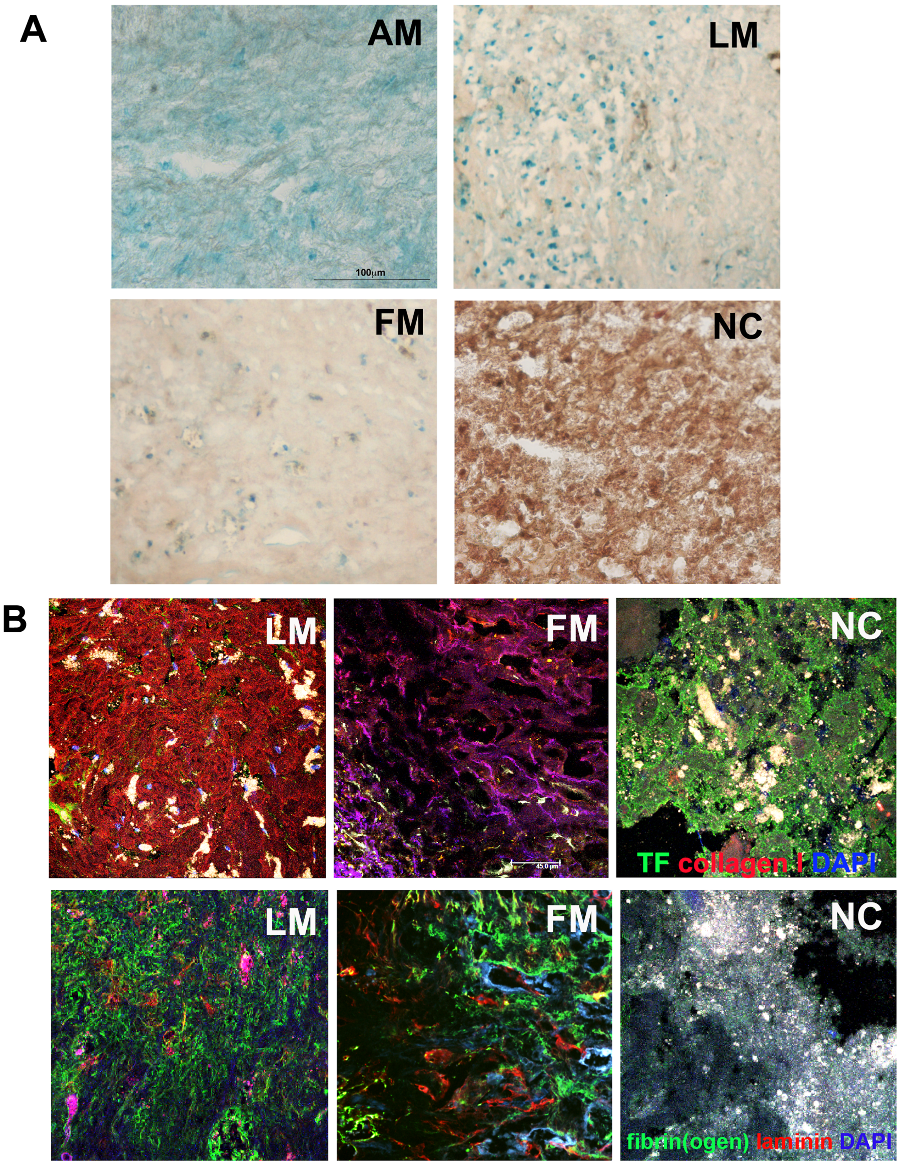

Supplemental Fig. 5 Thrombus formation over the CPL Media subtypes.
Media subtypes histological features (Movat's pentachrome stain) (**A**) and expression of TF, collagen type I, fibrin(ogen) laminin in confocal microscope representative fields (**B**) with the same X, Y than one of those quantified in blood flow experiments are shown in serial cryosections. Different markers' expression characterises the Media subtypes: fibro-calcific areas (FM), necrotic core (NC), areas rich in lipoproteins and/or foam cells (LM), or minimally altered areas (AM).

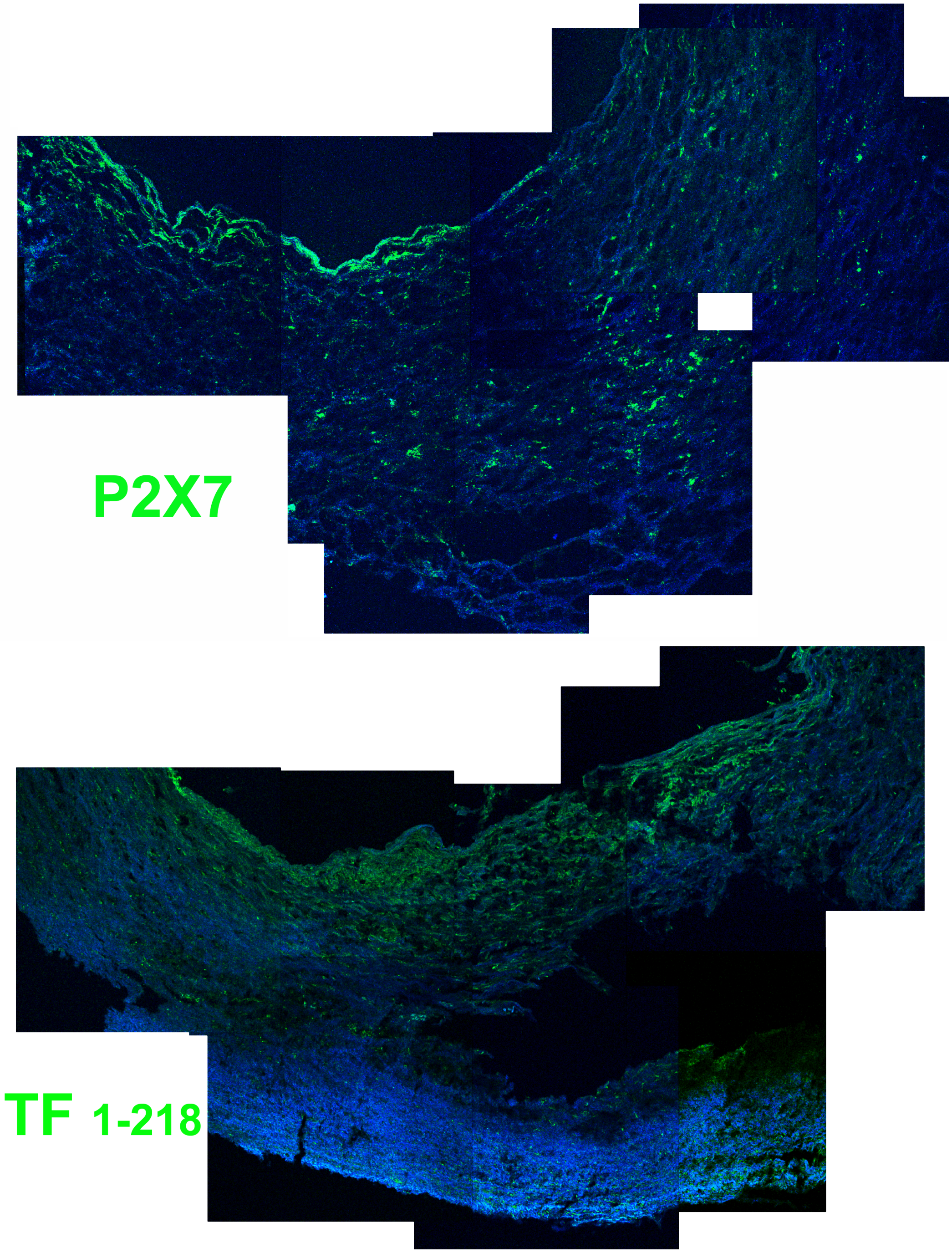

Supplemental Fig. 6 localization of P2X7 and TF over CPL
Partial reconstructions of 2 serial sections from a CPL, i.e. collages of 2D projection max images from confocal microscopy Z-series of adjacent fields, showing the distribution of P2X7 and of tissue TF are presented (single fields acquired with objective 40x)

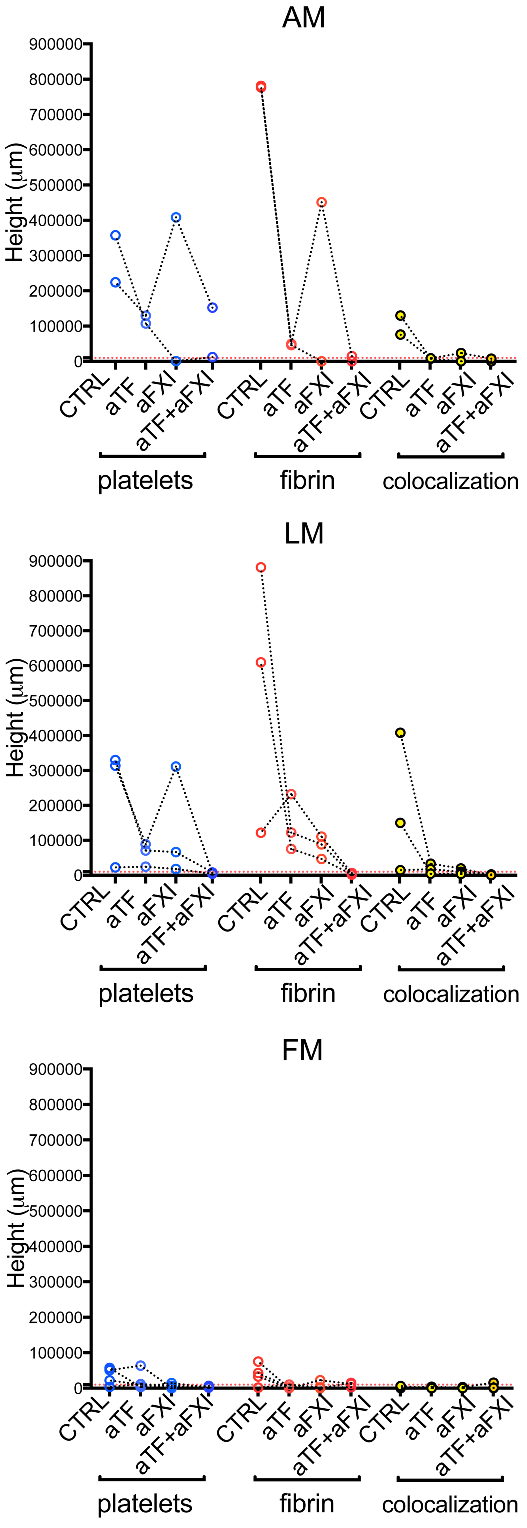

Supplemental Fig. 7 Thrombus formation over the CPL Media subtypes: corresponding fields
Quantifications in blood flow experiments of representative corresponding fields, i.e. with the same X, Y with respect to a repere, are shown for each Media subtype, in all conditions of antibody addition to blood. Data from single fields are presented as before-after dot plot with dotted lines connecting the fields with the same X, Y. Heights of platelet only, fibrin only and colocalized platelets with fibrin are shown, displaying that antibodies dramatically diminished colocalization.

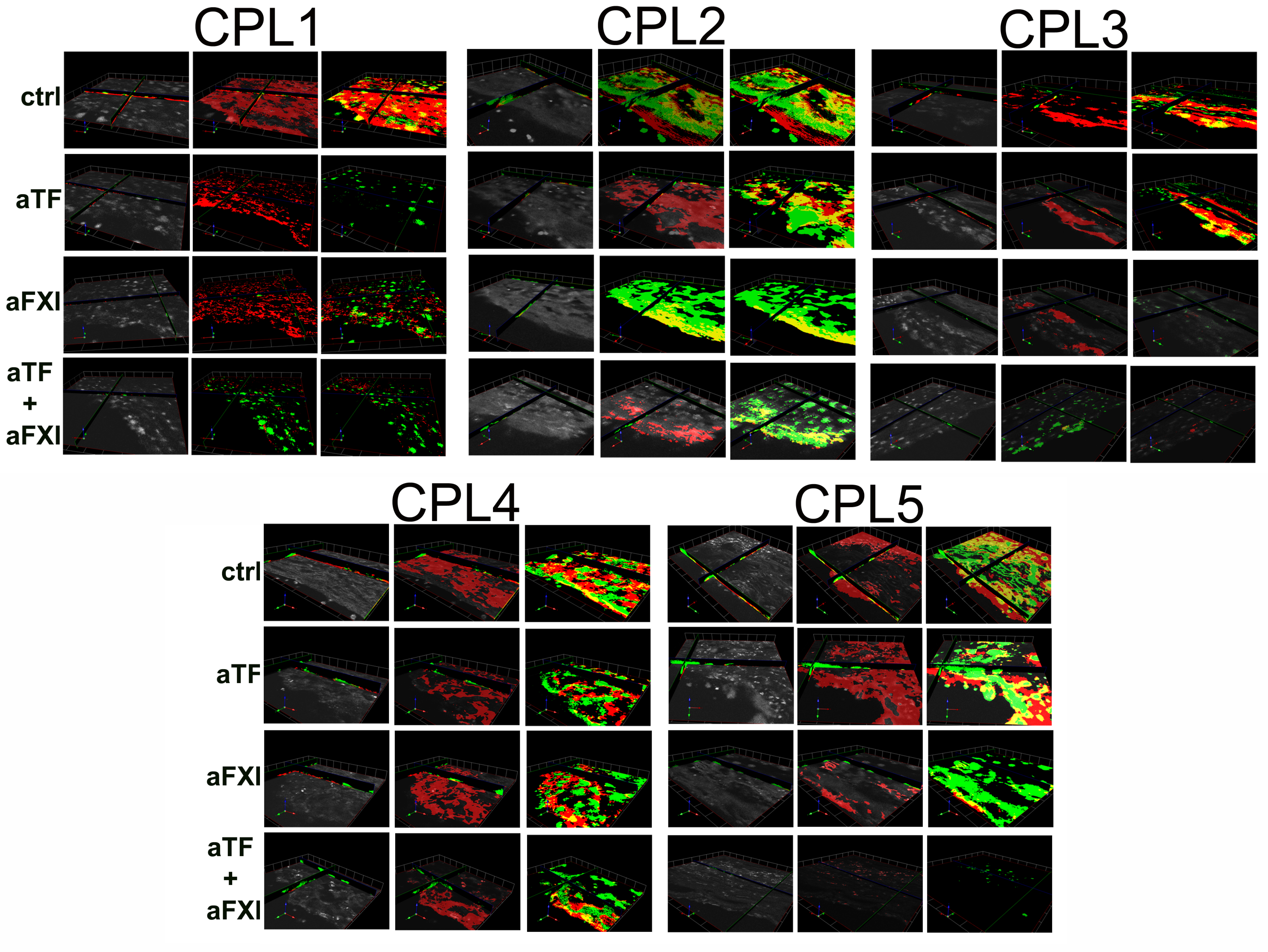

Supplemental Fig. 8 Thrombus formation over portions of CPL Intima
Representative XYZ planes obtained from colorized Z-sections from the last plane with tissue only, the first plane with fibrin and or platelets and an upper plane with both fibrin an platelets aggregates from corresponding fields perfused with blood with/without aTF and/or aFXI showing the pattern of thrombi distribution over Intima of five CPL.

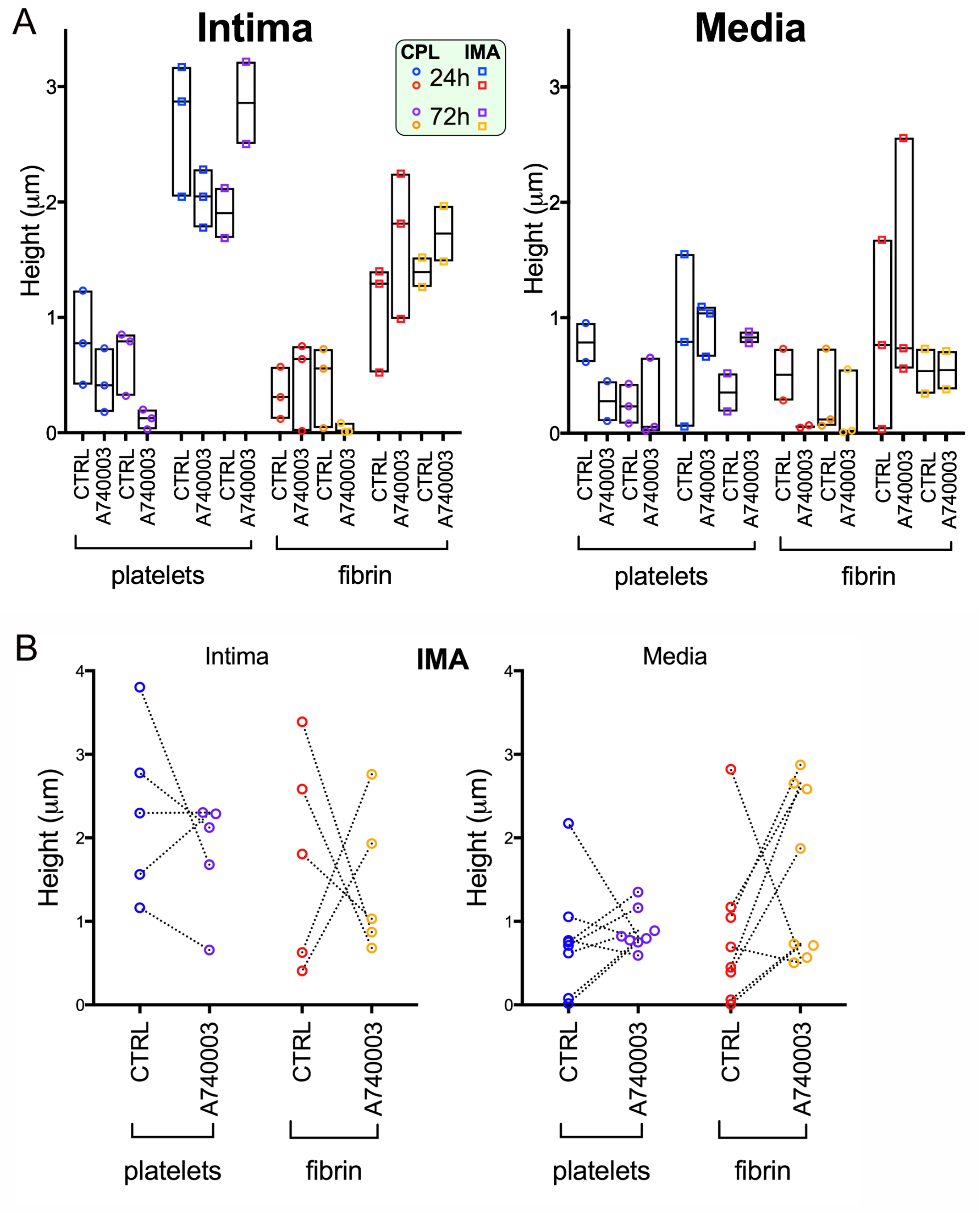

Supplemental Fig. 9 Effect of antagonism of P2X7 by A740003 on thrombus formation over IMA and CPL Intima. Quantification by confocal sectioning of platelet aggregates and fibrin over Intima and Media of CPL and IMA pre-treated with A740003/untreated either for 24h or 72h before sectioning and control blood perfusion (**A**). Data are presented as boxes (5-95 percentile) and dots indicate the mean value from 3-5 fields/sample; values showing thrombus height (volume/tissue area) are presented.

Quantitative analysis of thrombus formation over corresponding fields of Intima (**B, left**) and Media (**B, right**) of IMA pre-treated ex-vivo with A740003, sectioned and perfused with control blood showing the abscence of an inhibitory effect on platelets aggregation and fibrin strands deposition.

Supplemental Fig. 10 Summary of results on thrombus formation over CPL sections
